# HOPE AND RESILIENCE AMONG VULNERABLE, COMMUNITY-DWELLING OLDER PERSONS

**DOI:** 10.1093/geroni/igz038.1953

**Published:** 2019-11-08

**Authors:** Dennis R Myers, Clay Polson, Jocelyn S McGee, Rachel Gillespie

**Affiliations:** 1 Garland School of Social Work-Baylor University, Waco, Texas, United States; 2 Baylor University, Waco, Texas, United States

## Abstract

Community-dwelling older adults in the U.S. are at risk for experiencing a number of physical, emotional, and social issues including poverty, social isolation, and deteriorating health and daily functioning. Despite such challenges, research indicates that many older adults remain resilient and that factors such as social support, spirituality, and self-esteem contribute to resilience and improved outcomes. One factor that has been found to be particularly important for resilience among older adults is a sense of hopefulness. However, research has not looked specifically at the effects of hope on older adults experiencing severe economic and psychosocial challenges. Utilizing survey data drawn from a unique sample (n = 64) randomly drawn from 224 clients of a Meals on Wheels program, we explore the relationship between hope and resilience among a group of at-risk, community-dwelling older adults in one central Texas community. We find that hope, after accounting for the effects of social support, spiritual experience, health (ADL), and ethnicity, is a strong and significant predictor of resilience among at-risk older adults and that hope tends to mediate the effect of spiritual experience on resilience. Drawing on these findings, we discuss potential implications for social workers and congregational leaders working with older adults and for future scholarship on hope and resilience.

